# Oral Cysticercosis in a Pediatric Patient: A Rare Case Report with Review

**DOI:** 10.5005/jp-journals-10005-1355

**Published:** 2016-06-15

**Authors:** Puneet Goenka, Aditi Sarawgi, Kirti Asopa, Parvind Gumber, Samir Dutta

**Affiliations:** 1 Associate Professor, Department of Pediatric and Preventive Dentistry, Mahatma Gandhi Dental College, Jaipur, Rajasthan, India; 2 Senior Lecturer, Department of Prosthodontics and Crown and Bridge Mahatma Gandhi Dental College, Jaipur, Rajasthan, India; 3 Senior Lecturer, Department of Pedodontics, Mahatma Gandhi Dental College Jaipur, Rajasthan, India; 4 Senior Lecturer, Department of Oral Pathology and Microbiology, Mahatma Gandhi Dental College, Jaipur, Rajasthan, India; 5 Senior Professor, Department of Pedodontics, Government Dental College Rohtak, Haryana, India

**Keywords:** Cysticercosis, Parasite, *Taenia solium*, Tongue.

## Abstract

Cysticercosis is a condition in which a human acts as the intermediate host of the pork tapeworm *Taenia solium.* Although cysticercosis is a common disease in some regions of the world and can occur in any body site, oral lesions are rare. In this report, we document the case of oral cysticercosis in a 10-year-old boy who sought treatment for an asymptomatic nodule on the dorsal surface of the tongue. A detailed history, thorough clinical examination, morphological appearance and the histopathologic findings of the excised cyst formed the basis for the diagnosis of the lesion.

**How to cite this article:** Goenka P, Sarawgi A, Asopa K, Gumber P, Dutta S. Oral Cysticercosis in a Pediatric Patient: A Rare Case Report with Review. Int J Clin Pediatr Dent 2016;9(2):156-161.

Cysticercosis is a condition in which humans act as the intermediate host of *Taenia solium,* a pork tapeworm. The life cycle of *T. solium* is characterized by different stages of development, requiring various kinds of hosts that can appropriately harbor the eggs (proglottids), the oncospheres, the larvae and the adults.^1,2^ Cysticercosis in humans is common in the cerebral tissue, subcutaneous tissue, muscle and the eye.^2^ The pathological conditions manifested are usually the functional disturbance of the infected tissue such as seizure and visual impairment.^3^ The oral cavity is a rare site of involvement by cysticercosis, even in an endemic area.^4,5^ In addition, cysticercosis presenting as a nodule or mass in the tongue is even more rare.^6^ A correct and precise clinical diagnosis is infrequently established and often confused with other benign lesions of the oral cavity.^5,7^ We report here a case of oral cysticercosis that was diagnosed based on the clinical findings, and the morphological and histopathologic appearance of the lesion.

## CASE REPORT

**Fig. 1:**
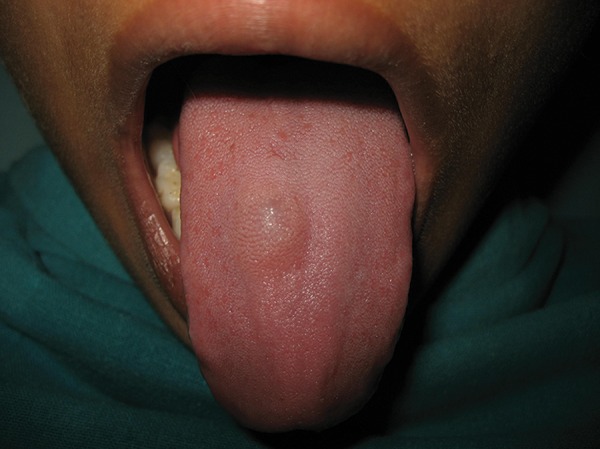
Swelling on the dorsal surface of the tongue

A 10-year-old boy reported to the dental clinic with a swelling on the tongue (Fig. 1). The lesion appeared around 3 years previously as a small localized swelling on the dorsal surface of the tongue, which had increased over the period to the present size. On examination, it was found to be 1.5 × 1.5 cm in size, oval, firm, nonmobile with a nonulcerated surface. The patient had no pain but had difficulty in eating. No significant history of fever was reported and the medical history was noncontributory. Mucocele, benign tumors of mesenchymal origin, such as lipoma, fibroma, hemangioma, lymphangioma, granular cell tumor, parasitic cyst and minor salivary gland adenoma were included in the differential diagnosis of this lesion. Fine needle aspiration cytology (FNAC) of the lesion was performed using a 22-gauge hypodermic disposable needle and a 5 ml disposable syringe. Around 1 ml of clear fluid was collected which showed some pearly white flakes. Smears were prepared and stained with May-Grunwald-Giemsa stain and hematoxylin & eosin stains and sent for cytological examination. Microscopic evaluation showed a mixed inflammatory reaction with numerous eosinophils, plasma cells and palisading histiocytes.

**Fig. 2:**
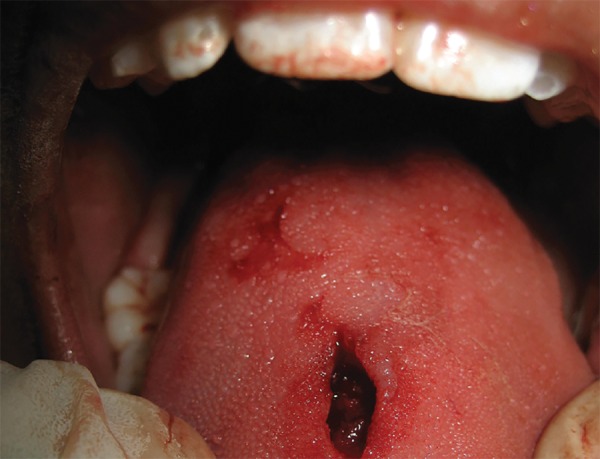
Surgical excision of the cyst

**Fig. 3:**
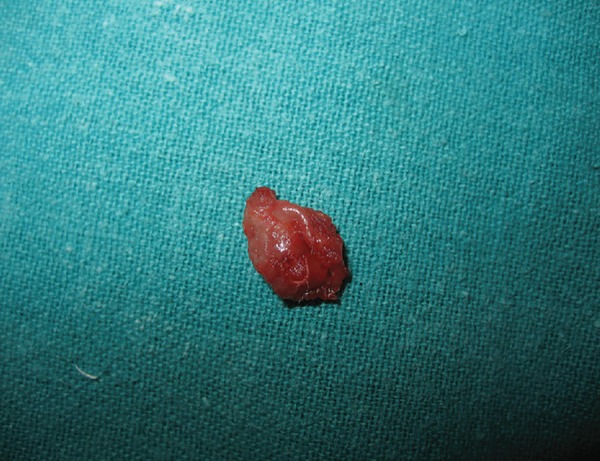
The enucleated cyst

**Fig. 4:**
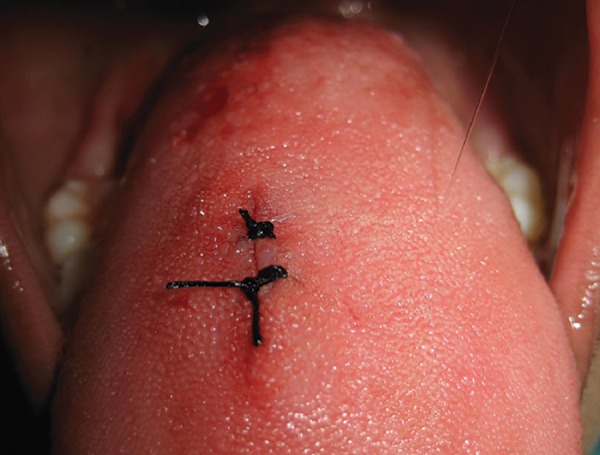
Sutures placed after surgery

**Fig. 5:**
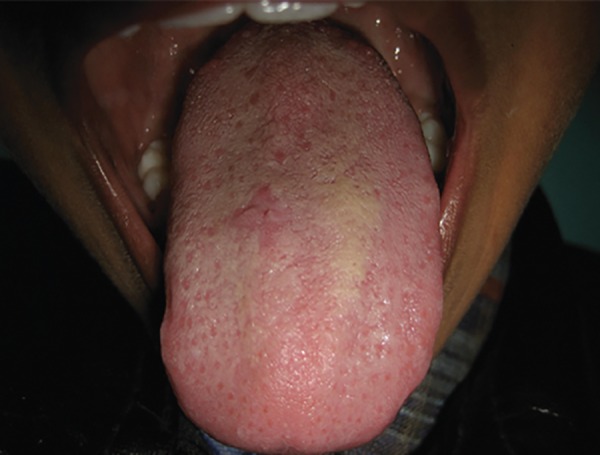
Healed tongue after surgery

**Fig. 6:**
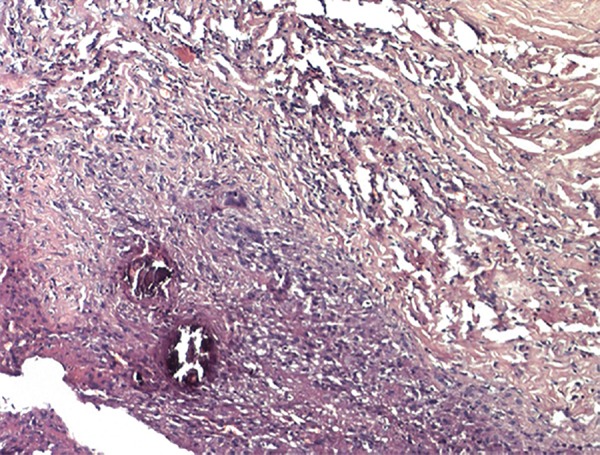
Photograph showing the histopathology of the excised cyst

The cyst was enucleated under local anesthesia and was sent for histopathologic examination (Figs 2 and 3). The incisional wound was sutured using silk suture material (Fig. 4). The surgical site showed an uneventful healing at 1 week postoperative examination (Fig. 5). Histopathology revealed an intramuscular cyst lined by palisaded histiocytes and encircled by fibrocollagenous tissue infiltrated by mixed inflammatory infiltrate including many eosinophils. Although a definitive parasite could not be identified, a small area of calcification was strongly suggestive of a healed parasitic cyst (Fig. 6).

The patient was referred to the Department of Pediatrics for thorough systemic examination. Computed tomography (CT) of the head and neck was found to be unremarkable. Stool, urine and blood failed to show active parasitosis. Oral antihelminthic drug was planned for the patient. To avoid any immune response to the parasitic byproducts, steroids were started first. Prednisolone 20 mg 8 hourly was administered orally for 5 days. On the 4th day, Albendazole 600 mg once daily was started and was continued for 28 days. The patient was monitored for 24 hours after administration of the first dose of Albendazole. The patient was scheduled for periodic examinations to assess his clinical status, which remained satisfactory for 3 years of follow-up.

## DISCUSSION

**Fig. 7:**
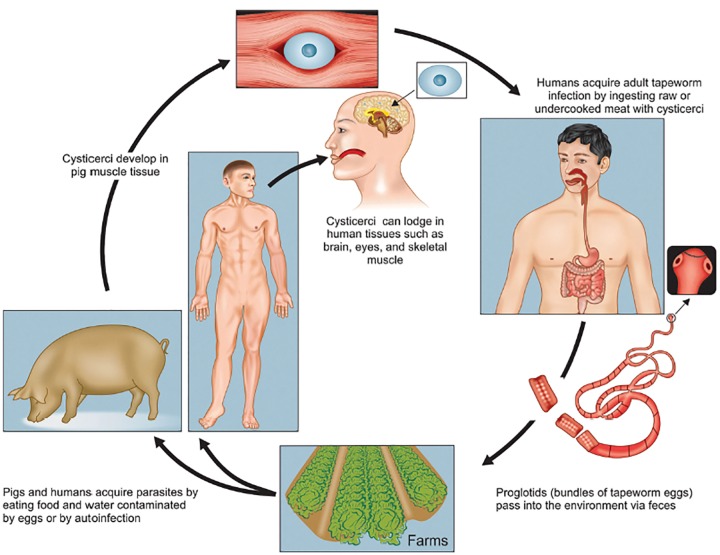
Photograph showing the life cycle of *Taenia solium*

*Taenia solium* (tape worm) is a hermaphrodite cestode for which human beings are the only definitive host. The adult worm is composed of the head (scolex) and numerous proglottids and may reach up to 6-10 ft. The proglottids (each containing 50,000-60,000 fertile eggs) are liberated by the humans in excreta. Cysticercosis develops when these eggs are ingested by humans and pigs (intermediate hosts) and oncospheres (embryos) are liberated by the action of gastric acid and intestinal juices. Infestation is usually via the oral route after consumption of contaminated food or drinks, or by unclean hands (feco-oral route) or, rarely, by reflux of the proglottid from the intestines into the stomach.^7^ The oncospheres and larvae that are formed in the stomach cross the bowel wall and actively reach destinations like brain, skeletal muscle, eye and subcutaneous structures through blood and lymphatics. Reaching these organs, the larvae become fluid-filled cysts known as the ’bladder worm’ or cysticerci.^8,9^ The pictographic description of the tapeworm life cycle has been shown in Figure 7.

Although cysticercosis is highly prevalent in some parts of the world (India, Indonesia, Africa, Peru and Mexico), oral and perioral lesions are relatively rare.^10^ In a large series of 450 cases, Dixon and Lipscomb^11^ detected oral involvement in only eight cases (1.8%). The condition in pediatric age group (0-18 years) involving the oral structures is even rarer. A review of few reported cases of cysticercosis in pediatric age group is shown in Table 1.

Although involvement of the tongue musculature by cysticercosis is common in swine, this location is rare in humans. No explanation for this phenomenon has been given, but some authors have suggested that the high muscular activity and metabolic rate of these muscles in humans might act against the lodgment and development of the cysticercus in this location.^12^ According to the literature, oral cysticerci usually elicit a clinical diagnosis of mucocele, or a benign tumor of mesenchymal origin, such as lipoma, fibroma, hemangioma, lymphangioma, granular cell tumor or a minor salivary gland adenoma.^1,4,13,14^ The lesion presented in this case as a firm nodule, thus making lipoma and hemangioma as less likely diagnosis. The consistency of lipoma is usually soft to fluctuant and histologically is composed of adipocytes that are subdivided into lobules by septet of fibrous connective tissue.^15^ Moreover, hemangioma presents as a flat or raised lesion, usually deep red or bluish which is often traumatized leading to surface ulcerations and secondary infections. Although tongue is the most common intraoral site for lymphangioma, it is a less probable diagnosis as they usually are present since birth or appear at a very early age. The histopathologic findings were also not in favor of either hemangioma or lymphangioma.

**Table 1:** **Table 1:** Reported cases of oral cysticercosis in pediatric age group

S. *No.*		*Age*		*Gender*		*Location*		*Author*	
1.		3		F		Tongue		Rao et al^31^	
2.		6		F		Upper lip		Hansen and Allard^32^	
3.		12		M		Lower lip		Hansen and Allard^32^	
4.		7		F		Tongue		Ostrofsky and Baker^33^	
5.		7		F		Buccal mucosa		Romero De Leon and Aguirre^2^	
6.		9		F		Submental area		Timosca and Gavrilita^34^	
7.		9		F		Buccal mucosa		Delgado-Azañero et al^10^	
8.		15		F		Buccal mucosa		Delgado-Azañero et al^10^	
9.		13		M		Lower lip		Delgado-Azañero et al^10^	
10.		18		M		Upper lip		Delgado-Azañero et al^10^	
11.		14		F		Upper lip		Delgado-Azañero et al^10^	
12.		6		M		Lower lip		Delgado-Azañero et al^10^	
13.		11		M		Tongue		Lustmann and Copelyn^35^	
14.		18		M		Lip (angle of mouth)		Nigam et al^14^	
15.		6		F		Tongue		Saran et al^7^	
16.		8		M		Tongue		Saran et al^7^	
17.		12		M		Tongue		Saran et al^7^	
18.		3		M		Tongue		Saran et al^7^	
19.		11		M		Buccal mucosa		Saran et al^7^	
20.		11		M		Submental		Mahindra et al^36^	
21.		7		F		Tongue and upper lip		Mukesh et al^37^	
22.		10		F		Tongue		Mukesh et al^37^	
23.		18		F		Upper lip		Mukesh et al^37^	
24.		5		F		Tongue, subcutaneous		Webb et al^38^	
25.		12		F		Tongue		Puppin et al^39^	
26.		12		F		Tongue		Gupta and Gupta^40^	
27.		11		M		Upper lip		Deshmukh et al^41^	
28.		10		M		Tongue		Goenka et al*	

*Current article

Very often the clinical presentation of granular cell tumors is very similar to the one presented in this case, i.e. a solitary, slow growing, painless, smooth and sessile mucosal swelling with a firm texture and color varying from normal or slightly pale to yellowish. The lesion can appear at any age with a peak age of 40-60 years and a female predilection of 2:1.^16^ Microscopically, granular cell tumors exhibit round or polygonal cells with small excentrically placed nuclei and abundant pale eosinophilic granular cytoplasm. The cells are usually arranged in unencapsulated sheets but may also be found in cords and nests. The histopathologic picture found in the presented case did not resemble the one described for granular cell tumor. Thus, granular cell tumor was also ruled out as the diagnosis for this case.^17-20^

Although larvae or its fragments were neither identified in FNAC nor in histopathologic examination of the postexcision specimen, based on clinical and morphological pointers the lesion was suggested to be a parasitic cyst. Aspiration of a clear fluid with white flakes, intramuscular site, mixed inflammatory response with predominance of eosinophils and plasma cells, presence of palisading histiocytes and calcified structures on microscopic examination were highly suggestive of an intraoral cysticercosis.

Oral cysticerci are firm nodules on palpation because of its high intraluminal pressure.^10^ In contrast to the severity of the disease in cerebral, ocular or cardiac sites, oral lesions are usually well tolerated; however, it is important to carry out a detailed study in every case to exclude the presence of the parasite in other sites. In order of frequency, the tissues affected by cysticercosis are subcutaneous layers, brain, muscles, heart, liver, lungs and peritoneum.^21^ The intensity of the signs and symptoms produced by cerebral cysticerci (headaches, acute obstructive hydrocephalus and epileptic seizures) depends on the number of invasive oncospheres present and their anatomic location. In some cases, the symptoms may even suggest the presence of a cerebral neoplasm.^22^ Iridocyclitis, secondary glaucoma and cardiac arrhythmias may also occur.^17^

Radiologic imaging, serology and tissue biopsy can be used to confirm a diagnosis of cysticercosis. Imaging techniques, in particular CT and magnetic resonance imaging (MRI), are of great value to diagnose cerebral cysticercosis.^23,24^ The immunodiagnosis of human cysticercosis can be achieved in sera, cerebrospinal fluid and saliva by either enzyme-linked immunosorbent assay (ELISA) or enzyme-linked immunoelectrotransfer blot.^25,26^ Enzyme-linked immunoelectrotransfer blot has a specificity and sensitivity superior to ELISA for the diagnosis of cysticercosis.^27^ Enzyme-linked immunoelectrotransfer blot for cysticercosis antibodies is highly sensitive in patients with multiple intracranial lesions, but it is less sensitive in patients with single or calcified lesions.^28^ Apparently, imaging techniques are more reliable than serological tests for the diagnosis of neurocysticercosis.^22^

Oral cysticerci are usually easy to excise and the prognosis is good. In all cases, simple surgical excision seems to be sufficient to ensure complete removal of the lesions without postoperative complications. Treatment of multiple cysts may be unnecessary in asymptomatic individuals after confirmation of diagnosis, but in every case a thorough clinical and epidemiological survey has to be done to identify the possible source and magnitude of the problem in a given community.^10^ In the present case also, a simple surgical excision of the lesion was done, which was well tolerated by the patient.

Albendazole is currently the drug of choice for the treatment of systemic cysticercosis. It is an imidazole with antihelminthic properties. It was first used to treat human neurocysticercosis in 1987.^28^ Praziquantel (isoquinoline) is a broad-spectrum antihelminthic, whose efficacy to treat parenchymal neurocysticercosis has been confirmed in several long-term follow-up studies throughout the world.^29^ Clinical trials for the treatment of neurocysticercosis have revealed that both Albendazole and Praziquantel reduce the number of cerebral lesions as demonstrated by serial MRI and CT scans.^30^ Considering the epidemic nature and the severity of the disease, a future therapeutic alternative to regulate the transmission of helminthic disease could be vaccination.

## References

[1]  Pinswasdi P, Charoensiri DJ (1997). Cysticercosis in labial tissue. Case report.. Aust Dent J.

[2] Romero De Leon E, Aguirre A (1995). Oral cysticercosis.. Oral Surg Oral Med Oral Pathol Oral Radiol Endod.

[3]  Tandon PN (1983). Cerebral cysticercosis.. Neurosurg Rev.

[4]  Nigam S, Singh T, Mishra A, Chaturvedi KU (2001). Oral cysticercosis - report of six cases.. Head Neck.

[5]  Martelli H, Melo Filho MR, Santos LAN (2006). Oral cysticercosis.. Braz J Oral Sci.

[6]  Pandey SC, Pandey SD (2005). Lingual cysticercosis.. Indian J Plast Surg.

[7]  Saran RK, Rattan V, Rajwanshi A, Nijkawan R, Gupta SK (1998). Cysticercosis of the oral cavity: report of five cases and a review of literature.. Int J Paediatr Dent.

[8]  Strickland GT (1984). Hunter’s tropical medicine.

[9]  Nash TE, Neva FA (1984). Recent advances in the diagnosis and treatment of cerebral cysticercosis.. N Engl J Med.

[10]  Delgado-Azanero WA, Mosqueda-Taylor AM, Carlos-Bregni R, Muro-Delgado RD, Diaz-Franco MA, Contreras-Vidaurre E (2007). Oral cysticercosis: a collaborative study of 16 cases.. Oral Surg Oral Med Oral Pathol Oral Radiol Endod.

[11]  Dixon HB, Lipscomb FM (1961). Cysticercosis, an analysis and follow up of 450 cases.

[12]  Sharma AK, Misra RS, Mukherjee A, Ramesh V, Jain RK (1986). Oral cysticercosis.. Int J Oral Maxillofac Surg.

[13]  De Souza PE, Barreto DC, Da Silva Fonseca LM, Batista de Paua AM, Silva E, Gomez RS (2000). Cysticercosis of the oral cavity: report of seven cases.. Oral Dis.

[14]  Amatya BM, Kimula Y (1999). Cysticercosis in Nepal: a histopatho-logical study of 62 cases.. Am J Surg Pathol.

[15]  Chidzonga MM, Mahomva L, Marimo C (2006). Gigantic tongue lipoma: a case report.. Med Oral Patol Oral Cir Bucal.

[16]  Nagaraj PB, Ongole R, Bhujanga-Rao BR (2006). Granular cell tumor of the tongue in a 6-year-old girl - a case report.. Med Oral Patol Oral Cir Bucal.

[17]  Sousa FB, Osterne RLV, Matos Brito RG, Alves APNN, Soares ECS, Costa FWG (2010). Oral granular cell tumor: a study of twelve cases in a Brazilian population.. J Clin Exp Dent.

[18]  Eguia A, Uribarri A, Gay Escoda C, Crovetto MA, Martinez-Conde R, Aguirre JM (2006). Granular cell tumor: report of 8 intraoral cases.. Med Oral Patol Oral Cir Bucal.

[19]  Tosios K, Rallis G, Vallianatou D, Vlachodimitropoulos D (2006). Yellow White tumor on the floor of the mouth.. Oral Surg Oral Med Oral Pathol Oral Radiol Endod.

[20]  Becelli R, Perugini M, Gasparini G, Cassoni A, Fabiani F (2001). Abrikossoff’s tumor.. J Craniofac Surg.

[21]  Wortman PD (1991). Subcutaneous cysticercosis.. J Am Acad Der-matol.

[22]  Miranda A (1993). Neurocysticercosis.. Am Fam Physician.

[23]  Richards F, Schantz PM (1991). Laboratory diagnosis of cysticercosis.. Clin Lab Med.

[24]  Puri V, Gupta RK (1991). Magnetic resonance imaging evaluation of focal computed tomography abnormality in epilepsy Epi- lepsia.

[25]  Flisser A, Plancarte A, Correa D (1990). New approaches in the diagnosis of *Taenia solium* cysticercosis and taeniasis.. Ann Parasitol Hum Comp.

[26]  Diaz JF, Verastegui M, Gilman RH, Tsang VC, Pilcher JB, Gallo C, Garcia HH, Torres P, Montenegro T, Miranda E (1992). Immunodiagnosis of human cysticercosis *(Taenia solium):* a field comparison of an antibody enzyme linked immunosorbent assay (ELISA).. Am J Trop Med Hyg.

[27]  Wilson M, Bryan RT, Fried JA, Ware DA, Schantz PM, Pilcher JB, Tsang VC (1991). Clinical evaluation of the cysticercosis enzyme-linked immunoelectro-transfer blot in patients with neurocysticercosis.. J Infect Dis.

[28]  Escobendo F, Penagos P, Rodriguez J, Sotelo J (1987). Albendazole therapy for neurocysticercosis.. Arch Intern Med.

[29]  Del Brutto OH, Sotelo J, Roman GC (1993). Therapy for neuro-cysticercosis: a reappraisal.. Clin Infect Dis.

[30]  Takayanagui OM, Jardim E (1992). Therapy for neurocysticercosis: comparison between Albendazole and Praziquentel.. Arch Neurol.

[31]  Rao PLNG, Radhakrishna K, Kapadia RD (1990). Cysticercosis of the tongue.. Int J Paediatric Otorhinolaryngol.

[32]  Hansen LS, Allard RHB (1984). Encysted parasitic larvae in the mouth.. J Am Dental Assoc.

[33]  Ostrofsky MK, Baker MA (1975). Oral cysticercosis: three case reports.. J Dental Assoc SA.

[34]  Timosca G, Gavrilita L (1974). Cysticercosis of maxillo-facial region.. Oral Surg Oral Med Oral Pathol.

[35]  Lustmann J, Copelyn M (1981). Oral cysticercosis: review of literature and report of two cases.. Int J Oral Surg.

[36]  Mahindra S, Daljit R, Sohail MA, Maheshwari HB (1981). Cysticercosis in the practice of otolaryngology.. Acta Otolaryngol.

[37]  Mukesh S, Kacker SK, Kapila K (1986). Cysticercosis of the oral cavity: a clinicopathological study of ten and a half years.. J Indian Dent Assoc.

[38]  Webb J, Seidal J, Correll WA (1986). Multiple nodules on the tongue of a patient with seizures.. J Am Dental Assoc.

[39]  Puppin D, Cavegn BM, Delmaestro D (1993). Subcutaneous cysticercosis of the tongue mimicking a tumour.. Int J Dermatol.

[40]  Gupta SC, Gupta SC (1995). Cysticercosis of the tongue.. ENT J.

[41]  Deshmukh Avadhani A, Tupkari JV, Sardar M (2011). Cysticercosis of the upper lip.. J Oral Maxillofac Pathol.

